# Phase II Trial of Angiotensin-(1-7) for the Treatment of Patients with Metastatic Sarcoma

**DOI:** 10.1155/2016/4592768

**Published:** 2016-11-08

**Authors:** Paul D. Savage, James Lovato, K. Bridget Brosnihan, Antonius A. Miller, W. Jeffrey Petty

**Affiliations:** ^1^Department of Medicine, Section of Hematology and Oncology, Wake Forest School of Medicine, Winston-Salem, NC, USA; ^2^Comprehensive Cancer Center of Wake Forest University, Wake Forest School of Medicine, Winston-Salem, NC, USA; ^3^Department of Biostatistical Sciences, Wake Forest School of Medicine, Winston-Salem, NC, USA; ^4^Department of Surgical Sciences, Center for Hypertension and Vascular Research, Wake Forest School of Medicine, Winston-Salem, NC, USA; ^5^Department of Cancer Biology, Wake Forest School of Medicine, Winston-Salem, NC, USA

## Abstract

*Background.* Angiotensin-(1-7) [Ang-(1-7)] is an endogenous antiangiogenic hormone with anticancer activity. In a phase I study of Ang-(1-7), two of three patients with metastatic sarcoma experienced disease stabilization. This phase II study examined clinical and biomarker outcomes for patients with metastatic sarcoma.* Methods.* Ang-(1-7) was administered by subcutaneous injection at a dose of 20 mg daily. If excessive toxicities occurred in the first cohort, a dose deescalation cohort was allowed. Blood samples were obtained to measure changes in biomarkers.* Results.* Treatment was well-tolerated and the dose deescalation cohort was not required. Plasma PlGF concentrations following treatment were not statistically significantly changed. A significant increase in plasma Ang-(1-7) was observed at 4 hours after injection. The median progression-free survival was 2.7 months (95% CI; 1.4 to 4.1 months), and the median overall survival was 10.2 months (95% CI; 5.3 to 18.3 months). Two patients with vascular sarcomas demonstrated prolonged disease stabilization of 10 months (hemangiopericytoma) and 19 months (epithelioid hemangioendothelioma).* Conclusions.* Ang-(1-7) at a dose of 20 mg daily was well-tolerated. This prospective phase II study failed to confirm the PlGF biomarker effect identified in the prior phase I study. Prolonged disease stabilization in hemangiopericytoma and epithelioid hemangioendothelioma may warrant further investigation.

## 1. Introduction

Sarcomas are a diverse group of malignant tumors that arise from mesenchymal tissues including bone and soft tissues. While a few subtypes of sarcomas have specific effective regimens, the majority of patients with advanced sarcomas receive regimens largely based on whether the tumor is of bone or soft tissue origin. For the majority of sarcoma patients, first-line treatments have involved anthracycline-based combination chemotherapy [1]. The doublet of gemcitabine and docetaxel has been studied and also has activity in the front-line setting [2, 3]. As key molecular pathways are elucidated, a number of targeted agents including antiangiogenic therapies are being tested in phase II clinical trials [4, 5]. To date only one such targeted agent, pazopanib, has FDA-approval for treatment of patients with metastatic sarcoma that has progressed after initial treatment [6].

Angiotensin-(1-7) [Ang-(1-7)] is a naturally occurring peptide with antiangiogenic properties [7–11]. Recently, Ang-(1-7) has shown anticancer activity* in vitro* and* in vivo* [12–14]. The effects* in vitro* are linked to the antiproliferative properties of this drug in vascular endothelial cells and reduced secretion of proangiogenic peptides from cancer cells [8–10, 13–15]. Reduced production of angiogenic hormones is triggered by reduced HIF-1*α* transcription* in vitro* [15].

A phase I trial using subcutaneous administration of Ang-(1-7) for the treatment of patients with advanced solid tumors was previously reported [16]. This study evaluated the maximum tolerated dose (MTD), toxicities, and pharmacokinetics of Ang-(1-7) when given subcutaneously once daily for five consecutive days on a 21-day cycle. This study identified 400 mcg/kg as the MTD for this schedule. Serious adverse events that were at least possibly related to Ang-(1-7) included deep vein thrombosis (400 mcg/kg dose level), stroke (700 mcg/kg dose level), and cranial neuropathy (700 mcg/kg dose level). The observed half-life at the MTD was very short (36 minutes) with considerable intra- and interpatient variability.

Clinical benefit, defined as disease stabilization for more than three months, was observed in two of the three patients with metastatic sarcoma treated in the phase I study. This included one patient with pleomorphic liposarcoma who had a 19% reduction in tumor measurements. Clinical benefit was associated with reduction in plasma levels of the proangiogenic hormone, placental growth factor (PlGF). This effect was statistically significant at several time points following Ang-(1-7) administration, with the greatest reduction observed 4 hours after treatment [15, 16].

The current phase II study sought to confirm and extend the findings from the phase I study by prospectively evaluating radiographic responses by RECIST and changes in PlGF following treatment in a larger cohort of patients with sarcoma. A continuous daily dosing schedule was selected based on the short half-life of the drug and the time course of biomarker changes observed during the phase I study.

## 2. Materials and Methods

### 2.1. Patient Eligibility

Patients were required to have a histologically or cytologically confirmed bone or soft tissue sarcoma that was metastatic or unresectable, with measurable disease. Patients had to have disease progression despite 1 or 2 prior treatment regimens with chemotherapy or targeted anticancer agents. Patients were required to be over 18 years of age and have an Eastern Cooperative Oncology Group (ECOG) performance status of 0–2. Patients could not be taking ACE inhibitors or angiotensin II receptor blockers at the time of enrollment. Prior use of these drugs was allowed. Required laboratory criteria at study entry included an absolute neutrophil count ≥ 1,500/*μ*L, platelet count ≥ 100,000/*μ*L, estimated creatinine clearance > 30 mL/min, total bilirubin < 2 mg/dL, and AST and ALT < 3 times the upper limit of normal.

Prior therapies (including chemotherapy, surgery, and radiation) had to be completed at least 4 weeks before enrollment. Patients were not eligible if they were pregnant or nursing. Women of child-bearing potential and men were required to agree to use adequate contraception (hormonal, double-barrier method of birth control, or abstinence) prior to study entry and for the duration of study participation.

### 2.2. Study Design

Ang-(1-7) was supplied by Bachem AG (Bubendorf, Switzerland) under good manufacturing protocol (GMP) conditions. Ang-(1-7) was dissolved in bacteriostatic water, filtered, and aliquoted into glass vials. Vials were stored frozen, and after thawing, vials were maintained at refrigerated temperatures for no more than two weeks.

Ang-(1-7) was administered by subcutaneous injection once daily continuously. One cycle was defined as a 21-day period. Toxicities were assessed weekly during the first cycle and on the first day of each subsequent cycle. In the absence of unacceptable treatment-related toxicity, patients continued treatment until disease progression.

This study was approved by the Institutional Review Board of Wake Forest University. It was registered in the National Cancer Institute PDQ Database and ClinicalTrials.gov as NCT00974545 and NCT01553539. Duplicate ClinicalTrials.gov listings were identified and subsequently linked. All patients were required to provide written informed consent.

### 2.3. Dose Cohorts

Two dose cohorts were planned. In the first cohort, twenty patients were treated at a dose of 20 mg daily which is similar to the weight-based dose of 400 mcg/kg identified as the MTD in the phase I study. Unlike the phase I study, this study utilized continuous daily dosing rather than five days every three weeks. A planned interim safety evaluation was performed after the enrollment of 10 patients in the first cohort. A second cohort of twenty patients was planned as a dose deescalation cohort in the event that excess toxicities were observed in the first cohort. Patients in this cohort would have received Ang-(1-7) at a dose of 10 mg daily if excessive toxicities had been observed in the 20 mg cohort. Safety events that would trigger dose deescalation included 3 strokes in any cohort or other evidence of excessive toxicity.

### 2.4. Patient Assessment

Tumor measurements were performed using RECIST version 1.1 prior to initiation of treatment and then every two cycles until disease progression or unacceptable toxicity. Toxicities were graded using the Common Terminology Criteria for Adverse Events, version 3.0. Dose limiting toxicities (DLT) were defined as grade 3 or greater toxicities that were felt to be at least possibly related to Ang-(1-7) treatment. In the event of a DLT, the dose of Ang-(1-7) was reduced to 10 mg daily.

### 2.5. Measurement of Drug and Angiogenic Hormone Levels

In the phase I trial, statistically significant changes in PlGF concentrations were identified 4 hours after the first Ang-(1-7) injection. To prospectively evaluate this biomarker, PlGF levels as well as VEGF, Ang-(1-7), Ang II, and Ang I were measured prior to the first dose, 4 hours after the first dose, and after 21 days of treatment in the phase II trial. Blood samples were immediately placed on ice and processed within 30 minutes of collection. For the angiotensin peptides, blood was taken in an EDTA containing tube with a cocktail of inhibitors to prevent* in vitro* generation or degradation of peptides, as previously described [17]. Plasma samples were stored at −80°C. Concentrations of Ang-(1-7) (developed by laboratory), Ang II (Alpco, Windham, NH), and Ang I (Peninsula, San Carlos, CA) were measured by the Hypertension Core Facility of Wake Forest University using three established radioimmunoassay methods [14]. Aliquots of plasma from the same time points were assayed by Aushon Biosystems (Billerica, MA) using Searchlight ELISA technology to quantify circulating vascular endothelial growth factor (VEGF) and placental growth factor (PlGF). Samples were blinded prior to shipping.

### 2.6. Statistical Methods

The study was designed with 87% power to detect a response rate by RECIST criteria of 10% in the study population. Frequency and severity of treatment-related toxicities were examined by cohort. Levels of biomarker expression over time were evaluated using nonparametric, Wilcoxon signed rank tests. All analyses were two-sided, and a *P* value < 0.05 was considered statistically significant. Analyses were performed using SAS v9.1.3 (SAS Institute, Cary, NC) and Stata v10.1 (StataCorp, College Station, TX) and were performed by the Core Biostatistics Facility of the Comprehensive Cancer Center of Wake Forest University.

## 3. Results

### 3.1. Patients

Twenty patients were enrolled between December 2009 and December 2011. Patient demographics are displayed in Table 1. Multiple subtypes of sarcoma were treated on the study, with the most common subtype of sarcoma being uterine leiomyosarcoma.

### 3.2. Adverse Events

Treatment on the current phase II study was generally well-tolerated. The adverse events are listed in Table 2. Two patients with preexisting headache disorders reported an increase in headaches which was felt to be possibly related to treatment. No strokes or other neurologic toxicities occurred. Only one DLT attributed as at least possibly due to treatment occurred. This was a grade 3 uncomplicated deep vein thrombosis (DVT) which was treated with anticoagulation.

Three deaths occurred, either on study or within 30 days of completing treatment. All three of these deaths were attributed to progressive cancer and were felt to be unrelated to the Ang-(1-7) treatment. Attribution for each of these was independently reviewed by an institutional clinical research oversight committee. Of the three patients who died during this trial, two patients developed respiratory failure from progressive lung metastases and one patient developed biliary obstruction with sepsis attributed to malignant obstruction of the common bile duct.

### 3.3. Pregnancy

One patient became pregnant while receiving Ang-(1-7) on this study, for which her use of the study drug was immediately suspended; at the time of discontinuation she had taken Ang-(1-7) for more than 1 year. After having an unremarkable fetal ultrasound, she delivered a healthy child, and her child is developing normally. She was felt to have likely benefited from treatment with prolonged stabilization of disease prior to becoming pregnant, and her disease demonstrated minimal change without requiring additional treatment for more than 2 years after discontinuing Ang-(1-7).

### 3.4. Clinical Outcomes

**Figure 1:**
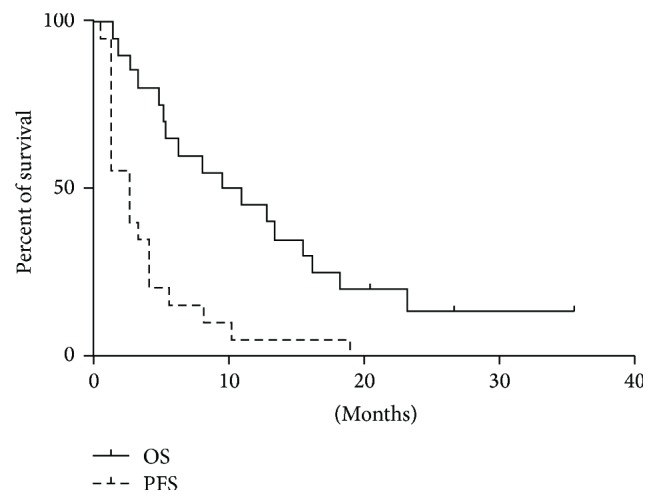
Kaplan-Meier graph is shown with progression-free survival (PFS) and overall survival (OS) for all patients enrolled in the study.

The study failed to achieve the primary endpoint of a 10% response rate. No partial or complete responses by RECIST were observed; however stabilization of disease for more than 3 months was observed in 9 of 20 (45%) patients. The median overall survival on this study was 10.2 months (95% CI; 5.3 to 18.3 months). The median progression-free survival on this study was 2.7 months (95% CI; 1.4 to 4.1 months). The progression-free and overall survival curves are shown in Figure 1.

Two patients with vascular sarcomas experienced prolonged periods of disease stabilization; both patients were demonstrating evidence of disease progression prior to study enrollment. One patient with epithelioid hemangioendothelioma exhibited an 18% increase in the size of lesions measured by the radiologist during a 5-month period of time prior to being enrolled in the study. This patient also developed a new liver lesion five months prior to enrollment. After starting treatment on study, the patient demonstrated stable disease by RECIST for 19 months. One patient with hemangiopericytoma demonstrated 2 new liver lesions as well as 22% increase size in lesions during a 3-month interval prior to enrollment. After starting treatment on study, the patient demonstrated stable disease by RECIST for 10 months. In the opinion of the treating physician, both patients showed evidence of clinical benefit due to a reduction in the pace of disease.

### 3.5. Biomarker Activity

**Figure 2:**
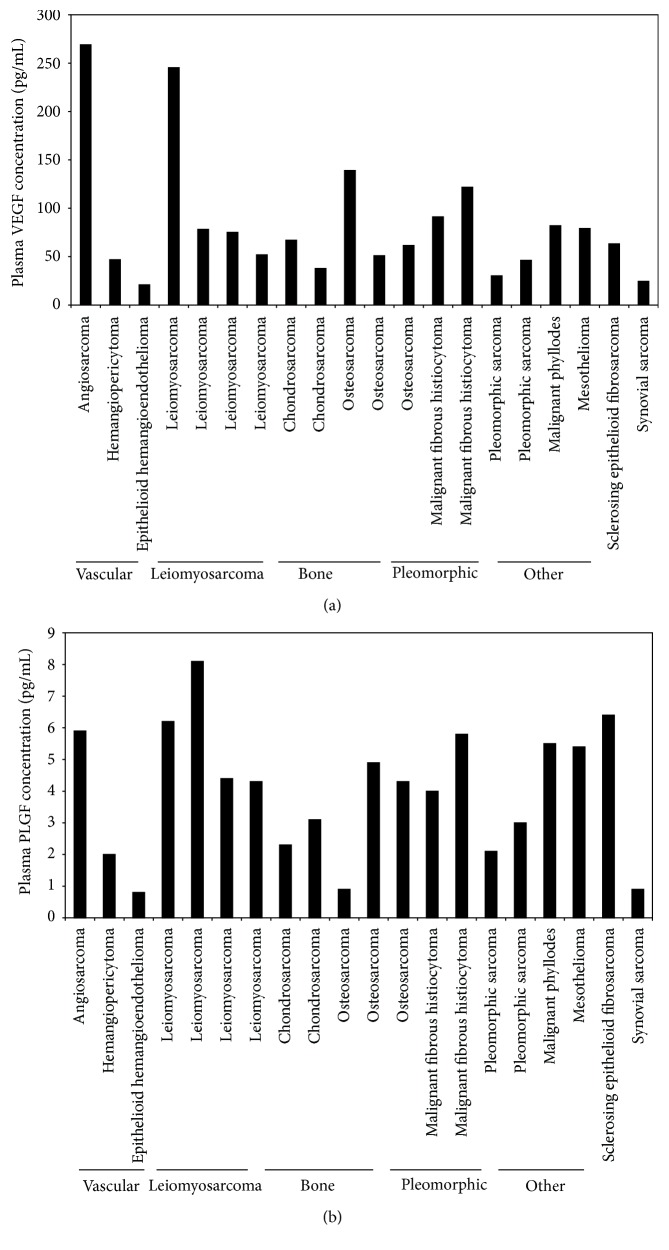
Pretreatment plasma concentrations of VEGF (a) and PlGF (b) are shown according to sarcoma subtype. Pretreatment VEGF concentrations were highest in the patient with angiosarcoma.

Concentrations of angiotensin system peptides and angiogenic hormones were measured prior to treatment and at two time points after treatment. The baseline concentrations of angiogenic peptides in plasma samples varied among the patients, as shown in Figure 2. Pretreatment VEGF concentrations were highest in a patient with angiosarcoma while PlGF concentrations demonstrated less variability across histologic subtypes.

**Figure 3:**
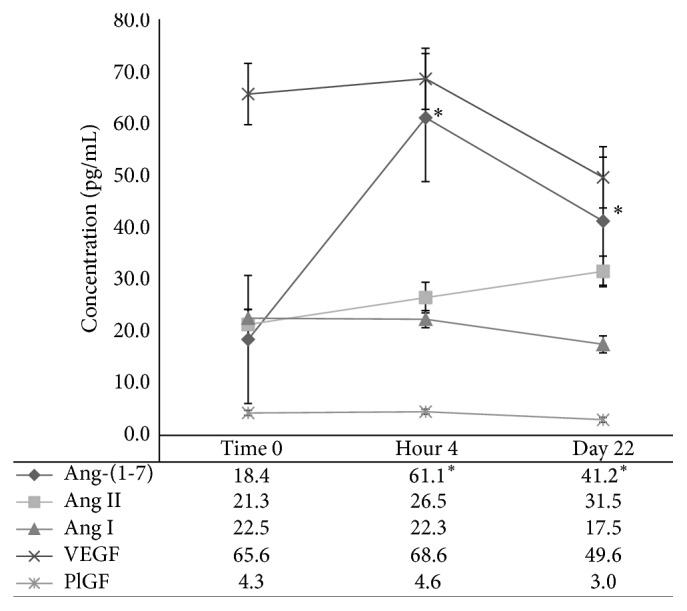
Plasma concentrations of angiotensin system peptides and angiogenic hormones over time are shown. The data points represent median values for the pretreatment (Time 0) and posttreatment blood draws. The 4-hour and day 22 posttreatment Ang-(1-7) levels were increased over baseline (*P* < 0.05). Standard error bars are shown.

Following treatment with Ang-(1-7), plasma Ang-(1-7) concentrations increased at the 4-hour and day 22 time points as shown in Figure 3. There were no changes in the levels of Ang I and Ang II following treatment. Significant changes were not observed in concentrations of VEGF or PlGF peptides although there was a nonsignificant trend to decreasing concentrations of both of these peptides on day 22. PlGF plasma concentrations for pretreatment, 4-hour, and day 22 time points were 4.3, 4.6, and 3 pg/mL. Changes in PlGF plasma concentrations were not statistically significant at these time points (*P* = 0.7 and *P* = 0.3).

## 4. Conclusions

Sarcomas are considered rare diseases for the purposes of drug development. In clinical practice, however, sarcomas are not a single rare disease but are instead a collection of extremely rare diseases with distinct cells of origin and differing sensitivities to treatment [1–3]. This presents a major challenge for drug development. Functional biomarkers that quantitatively measure biological effects are needed for facilitating clinical drug development decisions in rare and very rare diseases.

The clinical activity manifested by prolonged disease stabilization in our cases of hemangiopericytoma and epithelioid hemangioendothelioma may warrant further investigation in these two subtypes of vascular sarcoma. Other antiangiogenic compounds have been tested and have demonstrated some activity for these histologies [18–23]. The PFS for these two patients on our study of 10 months and 19 months compare favorably to the median PFS achieved with chemotherapy of 4.2 months [22].

Research regarding the anticancer properties of Ang-(1-7) is ongoing. Preclinical studies have shown compelling evidence of antiangiogenic effects using* in vitro*, murine wound healing [8] and murine xenograft models [13]. Our phase I study suggested a mechanistic role for PlGF in mediating its antiangiogenic effects. Although a trend towards decreasing PlGF and VEGF levels was observed on day 22 in the current study, these changes were not statistically significant.

A key pharmacologic barrier to the use of Ang-(1-7) is the short half-life. Stabilizing chemical modifications to the drug may be useful for overcoming this barrier. Alternatively, improved drug exposure could be accomplished by coadministration of an ACE inhibitor which has been demonstrated to inhibit the Ang-(1-7) degradation [24]. Similar chemical modifications and pharmacologic approaches have been explored to enhance the cardioprotective effects of Ang-(1-7) treatment [25–28]. The short half-life observed in the current study supports the exploration of these approaches for improving drug exposure and maximizing the anticancer activity of Ang-(1-7).

## Figures and Tables

**Table 1:** Patient characteristics.

Parameter	*N* (%) or mean ± SD (min–max)
Age (years)	55 (22–78)
Gender	
Male	11 (55)
Female	9 (45)

Race	
Caucasian	19 (95)
African American	1 (5)

Ethnicity	
Hispanic	2 (10)
Non-Hispanic	18 (90)

Sarcoma diagnosis	
Angiosarcoma	1 (5)
Chondrosarcoma	2 (10)
Epithelioid hemangioendothelioma	1 (5)
Hemangiopericytoma	1 (5)
Leiomyosarcoma	4 (20)
Malignant phyllodes	1 (5)
Mesothelioma	1 (5)
Osteosarcoma	3 (15)
Pleomorphic sarcoma (MFH/UPS)	4 (20)
Sclerosing epithelioid fibrosarcoma	1 (5)
Synovial sarcoma	1 (5)

Prior treatment	
Surgery	18 (90)
Radiation therapy	8 (40)
Chemotherapy	20 (100)

**Table 2:** Adverse events at least possibly related to treatment.

Toxicity	Grade			
	1/2	3	4	Total
Headaches	2	0	0	2
Fatigue	2	0	0	2
Muscle pain	1	0	0	1
Injection site reaction	6	0	0	6
Induration	1	0	0	1
Flushing	1	0	0	1
Flu-like symptoms	1	0	0	1
Pruritus	1	0	0	1
Deep vein thrombosis	0	1	0	1
Hyperbilirubinemia	1	0	0	1
Elevated alkaline phosphatase	1	0	0	1
Proteinuria	1	0	0	1

Total	18	1	0	19
